# Inhibitory and Inductive Effects of *Opuntia ficus indica* Extract and Its Flavonoid Constituents on Cytochrome P450s and UDP-Glucuronosyltransferases

**DOI:** 10.3390/ijms19113400

**Published:** 2018-10-30

**Authors:** Hyesoo Jeong, Soolin Kim, Mi-yeon Kim, Jimin Lee, Byoung Ha An, Hee-Doo Kim, Hyunyoung Jeong, Yun Seon Song, Minsun Chang

**Affiliations:** 1 Department of Biological Sciences, Graduate School of Sookmyung Women’s University, Seoul 04310, Korea; hyesoojeong@sookmyung.ac.kr (H.J.); soolinn@sookmyung.ac.kr (S.K.); petia13@naver.com (J.L.); abh0722@sookmyung.ac.kr (B.H.A.); 2 Department of Biology Education, College of Education, Sookmyung Women’s University, Seoul 04310, Korea; miy3638@sookmyung.ac.kr; 3 Department of Pharmacy, College of Pharmacy, Sookmyung Women’s University, Seoul 04310, Korea; hdkim@sookmyung.ac.kr; 4 Department of Biopharmaceutical Sciences, College of Pharmacy, University of Illinois at Chicago, Chicago, IL 60612, USA; yjeong@uic.edu; 5 Department of Biological Sciences, Research Institute for Women’s Health, Sookmyung Women’s University, Seoul 04310, Korea

**Keywords:** *Opuntia ficus indica*, flavonoids, drug metabolism, cytochrome P450, uridine diphosphate-glucuronosyltransferase, drug-herb interactions, menopausal women

## Abstract

*Opuntia ficus indica* (OFI) is grown abundantly in arid areas and its fruits are regarded as an important food and nutrient source owing to the presence of flavonoids, minerals, and proteins. The previous report that OFI exerts phytoestrogenic activity makes it plausible for OFI-containing supplements to be used as alternative estrogen replacement therapy. In the case of polypharmacy with the consumption of OFI-containing botanicals in post- or peri-menopausal women, it is critical to determine the potential drug-OFI interaction due to the modulation of drug metabolism. In the present study, the modulating effects on the hepatic drug metabolizing enzymes (DMEs) by OFI and its flavonoid constituents (kaempferol, quercetin, isorhamnetin, and their glycosidic forms) were investigated using the liver microsomal fractions prepared from ovariectomized (OVX) rats, human liver microsomes, and human hepatocarcinoma cell line (HepG2). As a result, the oral administration of extracts of OFI (OFIE) in OVX rats induced hepatic CYP2B1, CYP3A1, and UGT2B1. OFIE, hydrolyzed (hdl) OFIE, and several flavonols induced the transcriptional activities of both CYP2B6 and CYP3A4 genes in HepG2 cells. Finally, OFIE did not inhibit activities of cytochrome P450 (CYPs) or uridine diphosphate (UDP)-glucuronosyltransferases (UGTs), whereas hdl OFIE or flavonol treatment inhibited CYP1A2 and CYP3A1/3A4 in rat and human liver microsomes. Our data demonstrate that OFIE may induce or inhibit certain types of DMEs and indicate that drug-OFI interaction may occur when the substrate or inhibitor drugs of specific CYPs or UGTs are taken concomitantly with OFI-containing products.

## 1. Introduction

Drug-herb interactions (DHIs) refer to phenomena where the pharmacokinetic or pharmacological behavior of a certain drug is modulated by herb constituents that are orally coadministered with the drug. DHI is regarded as one of the critical clinical concerns in the population who have a tendency of concomitant consumption of herb and prescribed drugs [1]. One of the major mechanisms of DHIs is the induction or inhibition of drug metabolizing enzymes (DMEs) [1,2]. The liver is the primary organ of drug metabolism as it contains the majority of enzymes responsible for oxidation and conjugative reactions such as cytochrome P450s (CYPs) and uridine diphosphate (UDP)-glucuronosyltransferases (UGTs), which play significant roles in the first-pass metabolism of many orally administered drugs or xenobiotics [3]. Age-related changes in the liver can affect the capacity for drug metabolism and sex hormones are reported to influence the expression and activities of CYPs and UGTs [4,5,6]. Thus, DHI is an important clinical concern when herbal supplements and prescribed medicines are consumed concomitantly in the elderly, whose drug metabolism capacity is affected by factors such as aging and low level of sex hormones [7]. However, intensive studies in preclinical or clinical settings to investigate the effects of age, sex, or polypharmacy on DHI outcomes are scarce due to a lack of focus on DHIs and inherent difficulties in study execution in the elderly population.

**Figure 1:**
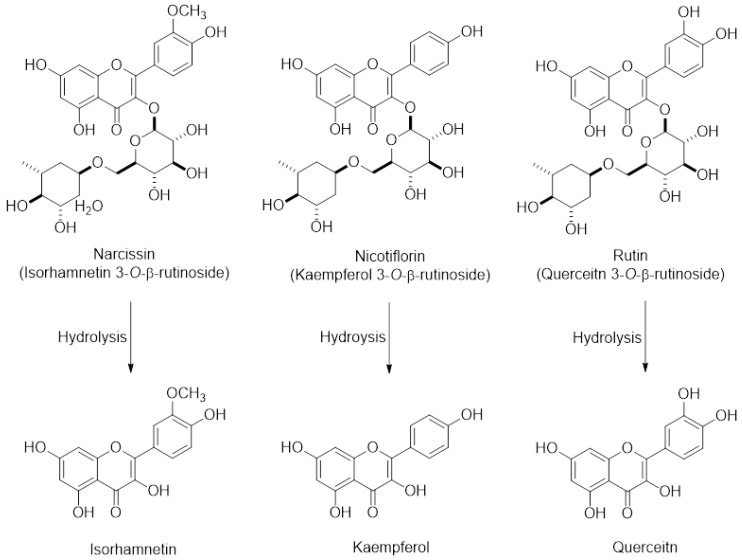
The chemical structures of major flavonoids in the forms of glycosides and their respective aglycones present in *Opuntia ficus indica* fruit extract (OFIE); Narcissin, nicotiflorin, and rutin are the 3-*O*-rutinosides of isorhamnetin, kaempferol, and quercetin, respectively.

*Opuntia ficus indica* (OFI) belongs to the Cactaceae family and grows abundantly in arid parts of the world such as South America and the Mediterranean area, and Southeast Asia such as Mexico and South Korea [8,9]. Fruits of OFI, commonly known as cactus pear or prickly pear, are regarded as an important nutrient and food source owing to phenolic antioxidants, fiber, minerals, and protein constituents. OFI fruits have been used for treating arteriosclerosis, diabetes, indigestion, inflammation, and other immune-related symptoms [10,11], implying that OFI-containing dietary products may be consumed by people who are likely to take medication to treat these conditions. In addition, we have previously demonstrated that the extract of freeze-dried OFI fruits (OFIE) can activate the estrogen receptor (ER)-mediated gene transcription in human cell lines and improve favorable lipid and glucose profiles in ovariectomized (OVX) rats [12]. The estrogenic activities of OFIE are attributed to the presence of flavonoid phytoestrogens such as isorhamnetin, kaempferol, quercetin, and their *O*-glycosides (Figure 1). These results suggest that OFI might be consumed as an alternative to estrogen-based menopausal therapy by the elderly females who are likely to take it concomitantly with other medicines. However, the safety profile of cactus pear under postmenopausal conditions has not yet been studied in terms of drug metabolism-mediated DHI. Hence, the objective of this study was to determine the effects of OFIE on both gene expression and function of major hepatic DMEs to predict the DHI potential of OFIE. In this study, we have investigated the inhibitory or inductive effects of OFI on major DMEs and the modulatory mechanisms of OFI using ex vivo rat liver tissue, subcellular fractions, and an in vitro human cell line. Our data indicated that specific isoforms of CYPs and UGTs are modified at the gene and/or activity levels in response to the treatment with the extract of OFI. Our results showed that induction of CYPs by OFI is mediated via interaction of transcription factors by the flavonoids present in OFI. In addition, the activities of some CYP isoforms were inhibited by the hydrolyzed (hdl) OFI and flavonols comprising the OFI constituents. Overall, our study provides valuable information on the safety of OFI use as a functional food or botanical supplements in postmenopausal women by determining the drug metabolism-mediated drug-OFI interactions.

## 2. Results

### 2.1. Inductive Effects of Oral Administration of OFIE on the Hepatic CYPs and UGTs Genes

The body weight ranged from 300 to 350 g at the end of the treatments compared to 220 to 250 g at the beginning of the experiments. There was no observable toxicities or abnormal behavior associated with the treatments (Supplementary information; The ARRIVE Guidelines Checklist).

**Figure 2:**
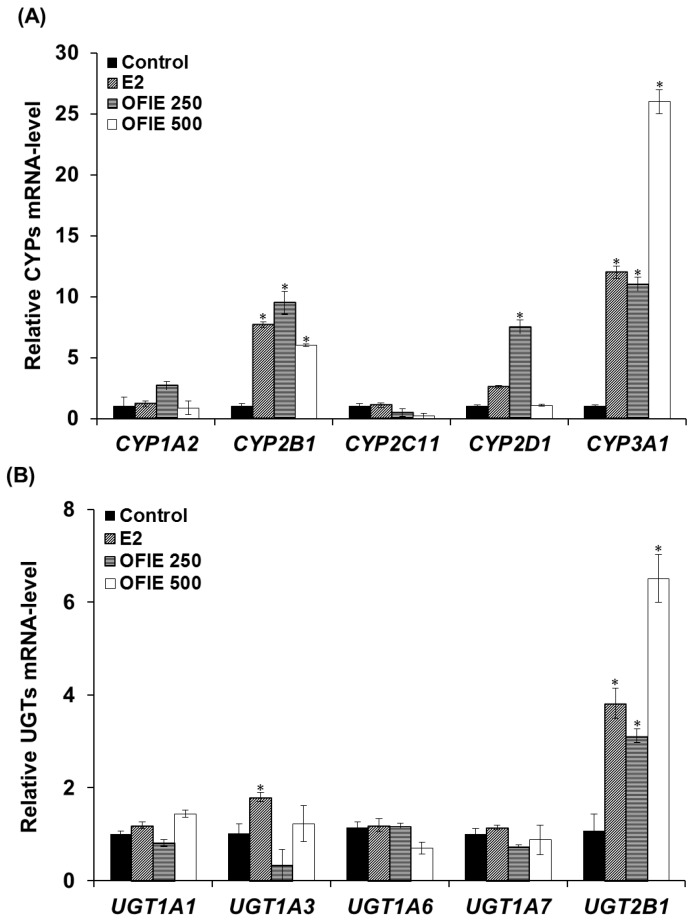
The mRNA expression levels of hepatic cytochrome P450s (CYPs) (**A**) and UDP-glucuronosyl transferases (UGTs) (**B**) in ovariectomized (OVX) rats after 5-week oral administration of *Opuntia ficus indica* fruit extract (OFIE). Each bar indicates the mean ± S.E. of triplicates. Control, the vehicle control-treated group; 17β-estradiol (E2, 0.5 mg/kg/day, positive control)-treated group; OFIE 250, (250 mg/kg/day)-treated group; and OFIE 500, (500 mg/kg/day)-treated group. Differences among gene levels in the various treatment groups versus those in the control group were determined with ANOVA followed by a Bonferroni-Dunn test. An asterisk indicates *p* < 0.001.

The mRNA levels of several major CYP and UGT isoforms in livers of OVX rats, which were subjected to the oral treatment of test compounds for 5 weeks, were quantified using a qPCR analysis. Treatment with 17β-Estradiol (E2) resulted in a 7.7-, 2.6-, and 12-fold increase in the mRNA expression of *CYP2B1*, *CYP2D1*, and *CYP3A1*, respectively (Figure 2A). OFIE treatment (250 mg/kg/day) resulted in a 3.1~12-fold increase in the expression of all CYPs except *CYP2C11* (Figure 2A). In rats treated with a high dose of OFIE (500 mg/kg/day), a 6.0- and 26-fold induction of *CYP2B1* and *CYP3A1* was observed, respectively; whereas the same treatment led to a significant decrease in mRNA expression of *CYP2C11* (Figure 2A). Among the five UGT isoforms investigated in our study, *UGT2B1* was the only gene with an increased expression (3.8–6.5 folds) after treatment with E2 and both doses of OFIE (Figure 2B).

### 2.2. Induction of Catalytic Activities of Liver Microsomal CYPs and UGTs by the Oral Administration of OFIE

**Figure 3:**
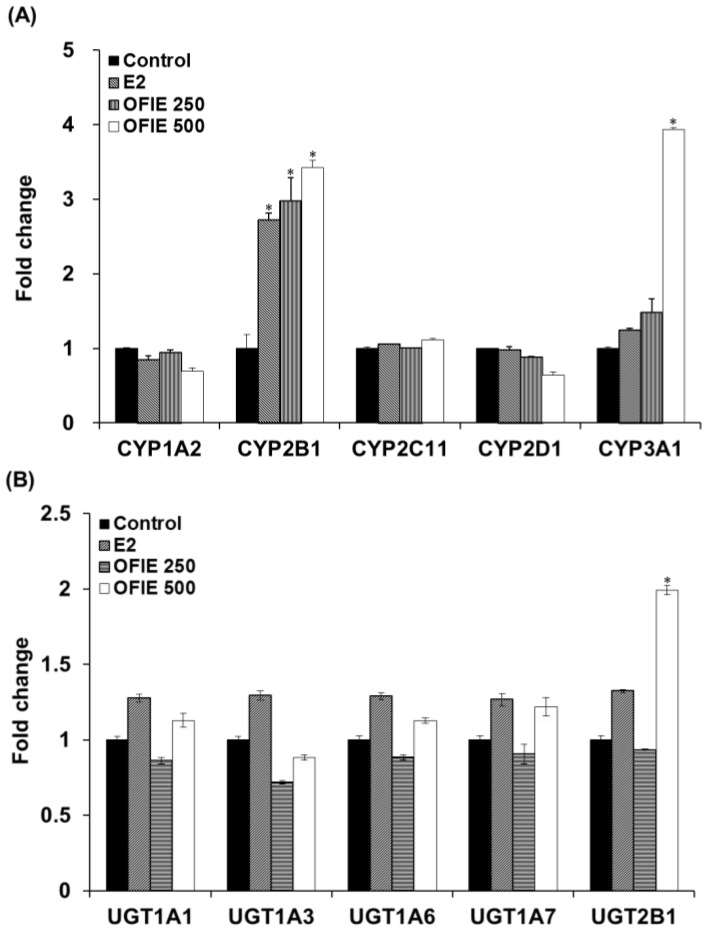
The relative amount of products formed in cytochrome P450 (CYP) (**A**) and UDP-glucuronosyltransferase (UGT) isozyme-catalyzed reactions (**B**) by liver microsomal fraction prepared from OVX rats, which were orally administered with E2 and *Opuntia ficus indica* fruit extract (OFIE) for 5 weeks. The amount of product was measured from a microsomal incubation containing CYP/UGT isozyme-specific substrate using high performance liquid chromatography (HPLC) or HPLC- tandem mass spectrometry (MS^2^) analysis. Values for fold change were obtained when the product formed from the microsomes of the control-treated group was set as 1. Control, olive oil-treated group; E2, 17β-estradiol (0.5 mg/kg/day)-treated group; OFIE 250, OFIE (250 mg/kg/day)-treated group; OFIE 500, OFIE (500 mg/kg/day)-treated group. Differences among products formed from each phenotyping reaction in the various treatment groups versus that in the control group were determined with ANOVA followed by the Bonferroni-Dunn test. An asterisk indicates *p* < 0.001.

The in vivo modulatory effects of OFIE on functions of DMEs were measured by means of ex vivo CYP and UGT activity assays in microsomal fractions prepared from the pooled livers of OVX rats. The E2 treatment resulted in a 2.7-fold increase in activities of CYP2B1 and no changes in activities all of UGT isoforms examined in this study (Figure 3A,B). OFIE treatment (250 mg/kg/day) increased the activity of CYP2B1 and CYP3A1 by 3.0-fold and 1.5-fold, respectively, with no effects on UGT activity. OFIE treatment (500 mg/kg/day) increased CYP2B1, CYP3A1, and UGT2B1 activity by 3.4-, 3.9-, and 2.0-fold, respectively (Figure 3A,B). CYP2C11 activity was significantly lower (67% and 80%, respectively) in rats treated with both doses of OFIE compared to that in rats treated with olive oil (Figure 3A).

### 2.3. Effects of OFIE on CYP2B6 and CYP3A4 Promoter Activities via CAR and PXR Transactivation

**Figure 4:**
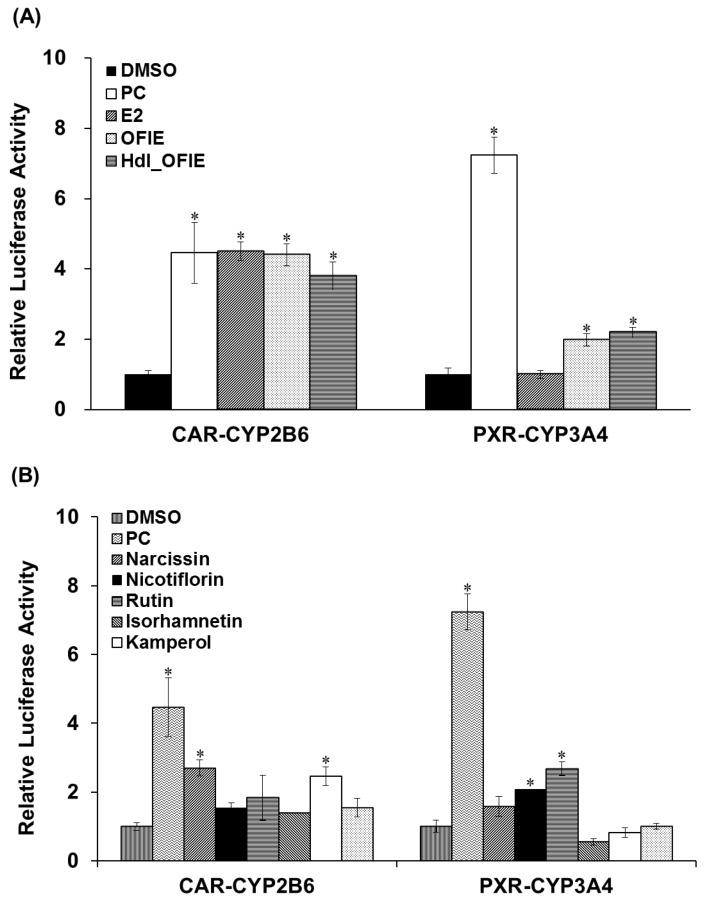
The transactivation of constitutive androstane receptor (CAR)-mediated CYP2B6 (**A**) and pregnane X receptor (PXR)-mediated CYP3A4 (**B**) promoter luciferase genes in HepG2 cells by *Opuntia ficus indica* fruit extract (OFIE) and its hydrolyzed product (A); and individual flavonoids (B). DMSO, dimethylsulfoxide (0.01%); PC, positive control, i.e., CITCO (500 nM) for CAR-CYP2B6 or rifampin (1 μM) for PXR-CYP3A4; OFIE, *Opuntia ficus indica* fruit extract (OFIE, 500 μg/mL), enzymatically hydrolyzed OFIE (Hdl OFIE, 500 μg/mL), and individual flavonoids (10 μM). Differences in the relative luciferase activities in various treatment groups versus that in the DMSO-treated group were determined with ANOVA followed by a Bonferroni-Dunn test. Differences in results among data sets were considered statistically significant when *p* < 0.001 indicated as an asterisk.

Promoter reporter assays were performed to investigate the effects of E2 or OFIE on *CYP2B6* or *CYP3A4* gene transcription via human constitutive androstane receptor (hCAR)- or human pregnane X receptor (hPXR)-associated mechanisms in HepG2 cells. OFIE is composed of flavonol aglycones and their *O*-glycosides (Figure 1) and cellulase and pectinase-mediated hydrolysis of OFIE leads to dramatic increases in the level of flavonol aglycones (Figures S1 and S2) [13], therefore, the ability of hdl OFIE to modulate CAR and PXR promoter activity was also investigated to speculate the roles for flavonoid constituents present in OFIE. E2, OFIE, and hdl OFIE induced a 4.8-, 4.4-, and 3.8-fold increase in CAR-mediated CYP2B6 reporter activity, respectively (Figure 4A). Both OFIE and hdl OFIE slightly induced PXR-mediated CYP3A4 reporter activity, whereas E2 did not alter CYP3A4 reporter activity. Six major flavonoids present in OFIE induced CAR promoter activity by >2-fold; whereas only glycosides, i.e., narcissin, nicotiflorin, and rutin, showed significant induction in the PXR promoter activity (Figure 4B).

### 2.4. Inhibition of Activities of CYPs and UGTs in Liver Microsomes by OFIE

**Figure 5:**
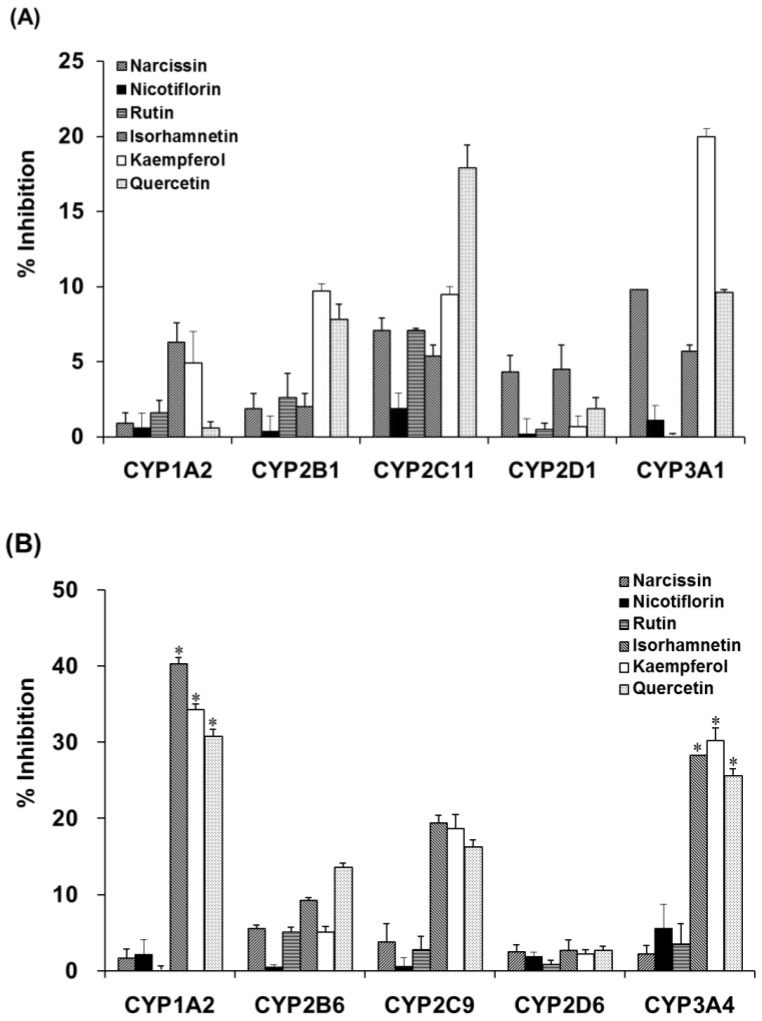
The inhibitory activity of cytochrome P450 (CYP) isozymes in liver microsomes from OVX rats (**A**) and pooled human liver microsomes (**B**) by six major flavonoid constituents present in *Opuntia ficus indica* fruit extract (OFIE). Percent inhibition was calculated when the enzyme activity was set as 100% in the incubation sample without any inhibitor. Differences among % inhibition of each phenotyping reaction in the various treatment groups versus that in the control group were determined with ANOVA followed by a Bonferroni-Dunn test. An asterisk indicates *p* < 0.001.

Incubation of liver microsomal fractions from OVX rats or humans with OFIE resulted in the non-significant inhibition of the activity of CYP (Table 1) or UGT (Table 2). However, enzymatic hydrolysis enhanced the inhibitory potency of the OFIE in rat CYP2C11 and CYP3A1 as well as human CYP1A2, CYP2C9, and CYP3A4 (Table 1). To confirm that the enhanced inhibition of CYP activity was due to flavonol aglycones in OFIE, whose amounts are increased by hydrolysis (Figure S2), individual constituents (10 μM) comprising OFIE were subjected to CYP inhibition assays. For rat CYP2C11 activity, quercetin and kaempferol showed 18% and 10% inhibition, respectively, whereas glycosides and isorhamnetin did not result in a significant inhibition (Figure 5A). Narcissin, quercetin, and kaempferol inhibited rat CYP3A1 by 10%, 10%, and 20%, respectively. In case of human CYP activity, isorhamnetin, kaempferol, and quercetin inhibited CYP1A2 by 40%, 34%, and 31%, respectively, whereas glycosides such as narcissin, nicotiflorin, and rutin did not significantly inhibit the CYP1A2 activity (Figure 5B). The three flavonol aglycones inhibited CYP2C9 and CYP3A4 activity by 4- to 10-fold more compared with the corresponding glycosides.

## 3. Discussion

In this study, the effects of OFIE on gene expression and the activity of major hepatic DMEs, i.e., CYPs, and UGTs, were examined in OVX rats and human ex vivo and in vitro models. Previously, OFIE has been demonstrated as a promising candidate for botanical menopausal hormone supplement owing to its phytoestrogenic activity [12]; therefore, an OVX rat model with conditions similar to the estrogen-deprived state of postmenopausal women was utilized to investigate the modulating effects of OFIE on DMEs and to determine the DHI potential in the present study. Furthermore, cultured cell line and cell-free liver extracts such as microsomal fraction were used as tools to investigate the inhibitory and inductive effects of OFIE or the constituents comprising OFIE.

Previous studies have demonstrated that the OFI fruit contains flavonoids, including rutin, narcissin, nicotiflorin, quercetin, kaempferol, and isorhamnetin as major phenolic compounds as well as vitamins, amino acids, and fibers [14,15,16]. Narcissin, nicotiflorin, and rutin glycosides are biotransformed into their respective aglycone such as isorhamnetin, kaempferol, and quercetin, respectively (Figure 1) [13]. Thus, the biological effects of OFIE are mainly attributed to flavonol aglycones rather than glycosides after oral consumption as aglycones are ready to trespass membranes [17]. The effects of quercetin, kaempferol, or herb extracts containing high amounts of these flavonols on DMEs have been studied using rat or human hepatocytes and immortalized liver or colon cells. These flavonols have been suggested to be inducers of rat CYP2B1 and human CYPs 1A2, 2B6, 3A4, and 2E1 [18,19] whereas our study showed that OFIE is an inducer of rat CYP2B1, CYP3A1, and UGT2B1 under estrogen-deficient conditions. The concentrations of flavonoids employed in the reported studies were in the μM range, which are approximately hundreds of fold higher compared to those of OFIE used in the present study. In addition, doses of flavonols utilized for in vivo studies ranged from 10 to 250 mg/kg/day [20], which are hundreds of folds higher compared to the estimated dose of flavonols derived from OFIE (20–40 μg/day) used in the present study [13]. Furthermore, studies conducted by other groups did not consider estrogen-deprived conditions and were performed in animals treated for a relatively shorter period (less than two weeks); whereas in the present study the duration of administration was five weeks. Regardless of the differences in experimental designs and the actual concentrations of flavonols used in various studies including our present study, it can be concluded that repeated oral administration of flavonols induces a certain type of DMEs. CAR and PXR are known as important regulators of DMEs, thus, CAR- and PXR-mediated coordination of DME expression is regarded as the principal mechanism of the body’s defense against toxic insults and the molecular basis for drug-herb/food interactions [21]. CAR binds to the phenobarbital responsive element module (PBREM), whereas PXR interacts with the xenobiotic response element (XRE) to activate the transcription of downstream target genes such as CYP2B6 and CYP3A4, respectively [22]. The inductive abilities of many herbal medicines and their bioactive ingredients on CYP2B6 and CYP3A4 have been studied using CAR or PXR-mediated PBREM or XRE reporter luciferase assays in HepG2 cells or primary human hepatocytes [23]. Our study demonstrated that OFIE and hdl OFIE are agonists for both hPXR and hCAR in HepG2 cells (Figure 4). It has been reported that isorhamnetin, kaempferol, and quercetin or flavonol-containing herbal extracts such as Ginkgo biloba extract (GBE) can lead to significant induction of CAR and PXR-mediated CYP2B6 and CYP3A4 transcriptional activities in HepG2 cells [24,25]. The composition of these three flavonols in a standardized GBE is very similar to that of OFIE [26], while the total amount of flavonols in OFIE was approximately six times lower than those in standardized GBE. This suggests that OFIE can exert an inductive effect on CAR or PXR-associated CYP2B6 or CYP3A4 promoter activities at a lower efficacy compared to GBE [24,25]. Although species differences exist between rodents and humans in substrate specificity and rate kinetics of drug metabolism, the hepatic expression levels of CYP2B and CYP3A in rats are also under the regulatory control of CAR and PXR, respectively, as seen in case of humans [27]. Thus, there exists the possibility of OFIE-mediated activation of CAR and PXR in OVX rats which lead to the induction of CYP genes as observed in the present study (Figure 2A,B).

**Figure 6:**
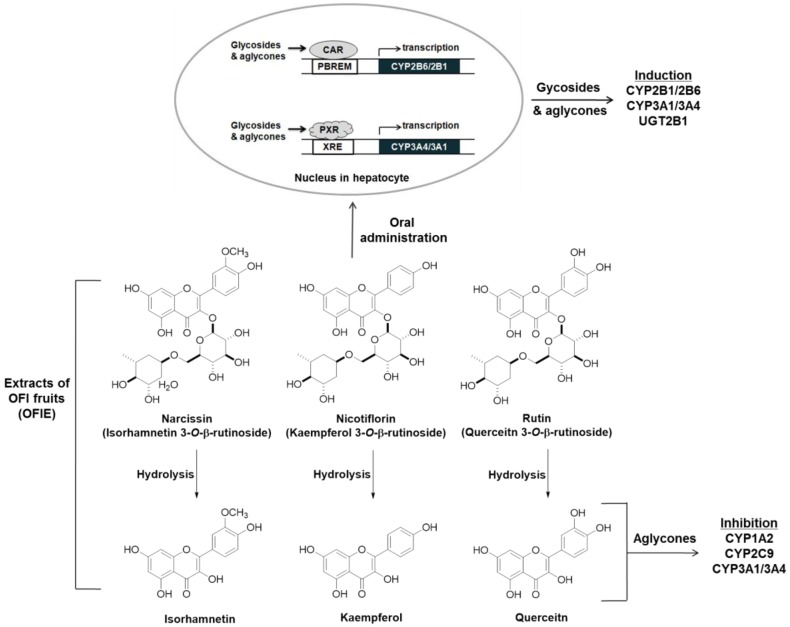
A scheme for the induction and inhibition of hepatic drug metabolizing enzymes (DMEs) by *Opuntia ficus indica* fruit extract (OFIE) and the related mechanisms. Narcissin, nicotiflorin, and rutin are glycosides, whereas isorhamnetin, kaempferol, and quercetin belong to the flavonol aglycone. Both glycosides and aglycones are involved in the induction of the expression of some CYPs and UGTs; on the other hands, only aglycones seem responsible for CYP inhibition.

Orally administered *O*-glycosides are susceptible to deglycosylation via hydrolytic enzymes in the liver or gut microflora, yielding aglycones that are more hydrophobic and smaller in size; these are considered the ultimate form of bioactive compounds in various types of biochemical processes such as CYP induction or inhibition [28]. In the present study, the ability of hdl OFIE rich in flavonol aglycones was investigated for the induction of CYP and inhibition of CYP and UGT. In addition, major flavonol aglycones present in OFIE, which increased as much as 10-fold via hydrolysis (Figures S1 and S2, and Table S3), were individually examined for their respective ability for enzyme induction or inhibition. Hdl OFIE induced CAR or PXR-associated CYP2B6 or CYP3A4 promoter activity in a manner similar to that of OFIE, implying the absence of significant differentiation in exerting transcriptional induction between aglycones and glycosides (Figure 6).

CYP or UGT inhibition serves as a pivotal mechanism for adverse DHIs. It is known that glycosides do not fit in the hydrophobic substrate pocket of CYPs either because of their bulky size or poor lipophilicity [29]. Von Moltke et al. reported that quercetin and kaempferol were inhibitors of CYP1A2 and CYP3A4 with IC_50_ values ranging from 10 to 16 μM; whereas flavonol glycosides did not affect the activity of these CYP isoforms [28]. In agreement with the study by Von Moltke et al. our study demonstrates that hdl OFIE rich in flavonol aglycones (Figure S2) induced significant inhibition of human CYP1A2 and CYP3A4, and rat CYP3A1. In addition, our result showed that flavonol aglycones were more potent inhibitors of rat CYP1A2, rat CYP3A4, human CYP1A2, and human CYP3A4 (Figure 5) than their corresponding glycosides. Upon the hydrolysis of glycosides in the gut, the aglycone metabolites are subject to absorption and distribution to the liver, where metabolism or modulation by enzymes occurs. Therefore, the aglycones that are originally present in OFIE and are formed as a result of interaction in the gut with the glycosides may play an ultimate role in drug-OFI component interactions in the liver. Our CYP and UGT inhibition studies were performed using OVX rat liver microsomes to obtain an insight into the effects of OFIE on DME activities under estrogen-deprived conditions. The human microsomal fraction purchased from Corning could not represent such conditions as it was prepared from pooled liver tissues donated by 150 individuals of mixed gender, age, and medical conditions. However, certain aspects of DHI potential of OFI in human could be predicted from our data. Finally, the inhibition of CYPs by hdl OFIE remained same when the microsomal fractions were incubated with hdl OFIE for 15 minutes prior to addition of cofactor (data not shown), implying that hdl OFIE is a competitive CYP inhibitor in rat or human liver microsomal systems.

The present study is the first attempt to investigate the DHI potential of OFIE under preclinical postmenopausal conditions and to extrapolate the results obtained from animal studies to human drug metabolism in vitro. Our results suggest that OFIE has the potential for DHIs by modulating hepatic DMEs at both gene and functional levels either through the induction of the genes or inhibition of catalytic activities. These modulatory effects exerted by OFIE are at least, in part, attributed to the flavonol aglycones as well as glycosides (Figure 6). Our study also indicates that the pharmacokinetic behavior of substrate drugs for CYP1A2, CYP2B6, CYP3A4, and UGT2B1 may be modulated when OFIE-containing supplements are taken concomitantly with these drugs by postmenopausal women. In the future, it is worthwhile that a more thorough pharmacokinetic study be performed to investigate the potential of drug-OFI interaction in preclinical models. Further studies would give clinically relevant information of drug-OFI interactions to ensure safer use of OFIE in the menopausal women.

## 4. Materials and Methods

### 4.1. Chemicals and Reagent

All chemicals and reagents were purchased from Sigma-Aldrich (St. Louis, MO, USA) unless stated otherwise. Narcissin and nicotiflorin were purchase from Extrasynthese (Genay, France). Solvents for HPLC experiments were purchased from Burdick & Jackson (Morristown, NJ, USA). All cell culture reagents were purchased from Gibco (Grand Island, NY, USA) unless stated otherwise. The Lyophilized powder of dried OFI fruits was purchased from Jeju Cactus Town (Hallim, Jeju-do, Korea) and a representative sample (150101MC) has been deposited at the Herbarium of the College of Science, Sookmyung Women’s University, Seoul, Republic of Korea. The composition of the OFI fruit extract has been analyzed using HPLC-diode array detector (DAD) as described previously [12,13] (Figure S1A and Table S3). Stock solutions of chemicals or OFI powder were prepared in dimethyl sulfoxide (DMSO) or olive oil and a solution of the lyophilized powder of OFI fruits was defined as OFIE in this study. The enzyme-hydrolyzed (hdl) OFIE sample was prepared as described previously [13]. The composition of the hdl OFIE has been presented in Figure S1B and Table S3. Human liver microsomal fraction (UltraPool^TM^ HLM150; Catalog No. 452117) was purchased from Corning (San Jose, CA, USA).

### 4.2. Animals

Female Sprague Dawley (SD) rats (8 weeks old, 200–220 g) were obtained from Orient Bio (Kyunggi, Korea) and housed in a specific pathogen-free facility at three animals per cage under standard animal laboratory conditions (21 ± 2 °C; 50–80% relative humidity; 12-h light/dark cycle). They were provided free access to water and a phytoestrogen-free rodent diet (Harlan, Host, The Netherlands). Animal studies were performed in accordance with the guidelines of the Korean Food and Drug Administration. The study protocol was reviewed and approved by the Institutional Animal Care and Use Committee of Sookmyung Women’s University (No. IACUC-1410-019 approved on 31 October 2014).

### 4.3. Ovariectomy, Oral Administration of Opuntia ficus indica Extract, and Preparation of Liver Microsomes

Rats were bilaterally ovariectomized under 1.5–3% isoflurane anesthesia as described previously [12]. After 14 days of endogenous hormonal decline, rats were randomly divided into several groups (*n* = 6 per group) and subjected to oral administration of olive oil (control), E2 (0.5 mg/kg/day, positive control) or OFIE (250 and 500 mg/kg/day) via gastric gavage for 5 weeks. OFIE doses were selected as 250 and 500 mg/kg/day since no significant acute or chronic toxicity was observed according to the general observation in animals treated with this dosage. Administration via forced gastric gavage was performed to ensure the exact amount of test compounds be dosed to each animal. E2 and OFIE were dissolved in olive oil and the volume of vehicle did not exceed 0.5 mL/day per rat. Animals were weighed daily and dosage was changed weekly according to weight gain. Animals were euthanized by carbon dioxide inhalation to minimize aversion or distress. Liver microsomal fractions were prepared from livers of OVX rats using differential centrifugation as described previously [30].

### 4.4. Quantitative Polymerase Chain Reaction (qPCR) Analysis 

The total RNA was isolated from homogenized rat liver tissue using a Nucleospin RNA/Protein kit (Macherey-Nagel, Düren, Germany) according to the manufacturer’s manual. The cDNA was synthesized from the total RNA (2 μg) using oligo (dT) and M-MLV reverse transcriptase (Promega, Madison, WI, USA). A qPCR analysis was performed to determine the mRNA levels of both hepatic CYP and UGT isozymes on an ABI Prism 7500 Sequence Detection System (Applied Biosystems, Foster City, CA, USA) using SYBR Green PCR Master Mix (Toyobo, Osaka, Japan) and a gene-specific primer set (Table S1 for primer sequences). The ABI 7500 Software (v2.0.6, Foster City, CA, USA) was used for the estimation of CT. The 2^−ΔΔ*C*t^ method was applied for relative quantification. The data were normalized against the geometric averages of expression levels for three internal genes; *GAPDH*, *Rplp1*, and *Rpl13a*. The results were expressed as fold change when the gene expression level in the vehicle control-treated sample was set as 1.

### 4.5. CYP and UGT Activity and Inhibition Assays 

CYP-mediated metabolite formation from a substrate (200 μM each) or the formation of glucuronide conjugates from the UGT isozyme-specific substrate (200 μM each) was monitored to determine the isozyme activity. Typical incubation was performed with rat (1 mg/mL) or human (0.5 mg/mL) liver microsomes in the presence of an appropriate cofactor in a buffer system (pH 7.4) at 37 °C for 60 min as described previously [31]. For inhibition studies, either OFIE or hdl OFIE as an inhibitor was included in the incubation mixture.

### 4.6. HPLC Instrumentation and Tandem Mass Spectrometry Analysis

The HPLC system (Agilent, Santa Clara, CA, USA) consisted of an Agilent 1200 binary pump, DAD, and Agilent 1260 autosampler. A Kinetex C_18_ reverse-phase column (5 μm, 4.6 × 150 mm) (Phenomenex, Torrance, CA, USA) protected by a KrudKatcher Ultra HPLC in-line filter (Phenomenex) was used for separation. HPLC conditions to determine the isozyme-specific metabolites and major constituents in OFIE are described in the Supplementary Materials. LC-electrospray (ESI)-tandem mass spectrometry (MS^2^) analysis was employed to detect glucuronide products. An Linear Trap Quadropole (LTQ) linear ion trap mass spectrometer (Thermo Scientific, Waltham, MA, USA) with an ESI source was coupled with an Agilent 1200 HPLC system. MS conditions used to detect glucuronides are summarized in Table S2.

### 4.7. Cell Culture

The human hepatocarcinoma cell line HepG2 was purchased from the American Tissue Culture Collection (Manassas, VA, USA) and maintained in Dulbecco’s modified Eagle’s essential medium (DMEM) containing 10% fetal bovine serum, 1% nonessential amino acids, 1% GlutaMAX, and 1% antibiotics/antimycotics in a 5% CO_2_ atmosphere at 37 °C.

### 4.8. Plasmid Transfection and Luciferase Reporter Assay

The expression plasmid for human constitutive androstane receptor (hCAR; pcDNA3-CAR) and pGL3-CYP2B6 luciferase construct were kindly provided by Dr. Hongbing Wang (University of Maryland, College Park, MD, USA). The plasmid for human pregnane X receptor (hPXR; pcDNA3-PXR) and pGL3-CYP3A4 luciferase construct were generous gifts from Masahiko Negishi (National Institutes of Health, Bethesda, MD, USA). HepG2 cells were seeded at a density of 1.5 × 10^5^ cells/well into a 24-well plate. On the next day, the cells were transfected with 0.25 μg of either pcDNA3-CAR or pcDNA3-PXR and 0.25 μg of promoter luciferase plasmid (i.e., either pGL3-CYP2B6 or pGL3-CYP3A4) using Lipofectamine 2000 (Invitrogen, Carlsbad, CA, USA) according to the manufacturer’s instructions. To normalize the transfection efficiency, the pRL-TK plasmid (0.25 μg/well; Promega), which contains cDNA encoding *Renilla* luciferase, was cotransfected. The transfected cells were grown for 24 h, treated with various test materials, and incubated for an additional 24 h. The luciferase activities in the cell lysates were measured using the dual luciferase assay system (Promega) with a SpectraMax i3x (Molecular Devices, San Jose, CA, USA). Data are reported as a relative luciferase activity, which is the firefly luciferase reading divided by the *Renilla* luciferase reading.

### 4.9. Statistical Analysis

Experiments using cells and microsomal incubations were performed in triplicate. Results are representatives of experiments repeated at least three times independently. The data were further analyzed using an analysis of variance (ANOVA) followed by a Bonferroni-Dunn test using Prism Version 3.0 (GraphPad Software, San Diego, CA, USA). Differences in results among data sets were considered statistically significant when *p* < 0.001. Significant difference is indicated by an asterisk in the figures, unless stated otherwise.

## Figures and Tables

**Table 1:** The CYP inhibitory effects of OFIE on CYP isozyme-mediated metabolism in OVX rat and human liver microsomes ^a^.

CYP Isozyme (Rat/Human)	Phenotyping Reaction	% Inhibition in OVX Rat Hepatic Microsomes ^a^		% Inhibition in Human Hepatic Microsomes	
		OFIE	Hydrolyzed (hdl) OFIE ^b^	OFIE	Hydrolyzed (hdl) OFIE
1A2/1A2	PCOD ^c^	16 ± 1.4 ^d^	22 ± 0.49	19 ± 2.3	41 ± 0.93
2B1/2B6	BPHY	4.3 ± 0.53	1.2 ± 0.44	5.8 ± 0.84	1.7 ± 1.4
2C11/2C9	TOLHY	0.7 ± 0.69	16 ± 0.55	6.8 ± 1.3	27 ± 1.4
2D1/2D6	DEXOD	16 ± 0.25	18 ± 0.53	5.5 ± 5.0	3.7 ± 6.8
3A1/3A4	TSTHY	0.70 ± 0.25	40 ± 0.43	19 ± 1.8	49 ± 2.0

^a^ The microsomal fraction was prepared from the livers of OVX rats treated with olive oil. ^b^ Hydrolyzed (hdl) OFIE refers to the OFI extract samples treated with hydrolytic enzymes (a mixture of cellulase and pectinase) prior to CYP inhibition assays. The methods used for the hydrolysis of OFIE are described previously [13]. ^c^ PCOD, phenacetin *O*-deethylation; BPHY, bupropion hydroxylation; TOLHY, tolbutamide 4-hydroxylation; DEXOD, dextromethorphan *O*-demethylation; and TSTHY, testosterone 6β-hydroxylation. ^d^ Numbers represent % of inhibition. Metabolite formation in the CYP-mediated reaction without OFIE samples was set as 100%.

**Table 2:** The inhibitory effects of OFIE on the activities of UGTs in OVX rat and human liver microsomes.

UGT Isozyme (Rat/Human)	Phenotyping Reaction	% Inhibition in OVX Rat Hepatic Microsomes		% Inhibition in Human Hepatic Microsomes	
		OFIE	Hydrolyzed (hdl) OFIE	OFIE	Hydrolyzed (hdl) OFIE
1A1/1A1	ESG ^a^	−1.6 ± 1.9 ^b^	−7.2 ± 1.9	16 ± 2.3	21 ± 2.9
1A3/1A3	CDCAG	2.9 ± 2.7	−5.0 ± 1.2	11 ± 0.9	16 ± 6.0
1A6/1A6	NPG	−4.7 ± 1.8	−9.7 ± 0.50	8.9 ± 1.6	7.5 ± 1.2
1A7/1A9	MPAG	7.7 ± 0.26	11 ± 2.8	8.2 ± 2.1	3.1 ± 4.9
Rat 2B1	TSTG	0.2 ± 1.9	−15 ± 0.97	N.A. ^c^	N.A.
Human 2B7	AZTG	N.A.	N.A.	11 ± 2.2	7.9 ± 3.0

^a^ ESG, 17β-estradiol 3-*O*-glucuronidation; CDCAG, chenodeoxycholic acid 24-glucuronidation; NPG, 1-naphthol β-d-glucuronidation; MPAG, mycophenolic acid *O*-glucuronidation; TSTG, testosterone β-d-glucuronidation; and AZTG, zidovudine 5′-glucuronidation. ^b^ Numbers represent % of inhibition. Metabolite formation in the UGT-mediated reaction without OFIE samples was set as 100%. ^c^ Not applicable.

## Supplementary Materials

Supplementary materials can be found at http://www.mdpi.com/1422-0067/19/11/3400/s1.
