# Supplementary material for: Inhibitory and Inductive Effects of Opuntia ficus indica Extract and Its Flavonoid Constituents on Cytochrome P450s and UDP-Glucuronosyltransferases
Source: Int J Mol Sci. 2018 Oct 30;19(11):3400. doi: 10.3390/ijms19113400 (PMC6274835; doi:10.3390/ijms19113400)
Supplement: Supplementary file 1 [file ijms-19-03400-s001.zip › ijms-361897-supplementary-final check/ijms-361897-supplementary-proofreading.docx]

Supplementary Materials

Methods

Preparation of Hydrolyzed OFIE

Freeze-dried OFI fruit powder was dissolved in water (100 mg/mL), sonicated for 20 min, and centrifuged at 10,000 × *g* for 10 min to remove water-insoluble fraction. The supernatant was filtered through a 0.45 μm-polytetrafluoroethylene (PTFE) syringe filter. The supernatant (500 μL) was subjected to hydrolysis at 50 °C for 2 h with an enzyme mixture of cellulase from *Aspergillus niger* (Sigma-Aldrich; 1.21 U/mg) and pectinase from *Aspergillus niger* (Sigma-Aldrich; 1.12 U/mg) at a unit ratio of 1:3.7.

HPLC Instrumentation

Mobile phase A consisted of water with 0.1% formic acid, whereas mobile phase B consisted of acetonitrile. The gradient was started with 10% B, increased to 90% B over 15 min, and then returned to the initial condition over 5 min. The solvent flow rate was set at 1 mL/min and the column temperature was set at 35 °C. Diode array detection (DAD) was set at a wavelength of 248 nm for phenacetin *O*-deethylation (PCOD), 298 nm for bupropion hydroxylation (BPHY), 276 nm for diclofenac 4′-hydroxylation (DCHY), 230 nm for tolbutamide 6-hydroxylation (TOLHY), 280 nm for dextromethorphan *O*-demethylation (DEXOD), and 250 nm for testosterone 6β-hydroxylation (TSTHY) and testosterone β-D-glucuronidation (TSTG), respectively.

Major flavonoids present in OFI extract were analyzed and quantitated using HPLC-UV. OFI powder (125 mg/mL) was dissolved in 70% methanol, sonicated for 1 h, and filtered through a 0.45-μm syringe filter. The filtrate (20 μL) was injected for analysis. The calibration curves for narcissin (Extrasynthese, Genay, France), nicotiflorin (Extrasynthese), rutin (Santa Cruz Biotechnology, Dallas, TX), quercetin (Santa Cruz Biotechnology), kaempferol (Sigma-Aldrich), and isorhamnetin (Santa Cruz Biotechnology) were generated based on the peak areas of standard samples at appropriate concentrations. Samples were run on Kinetex C18 columns (5 μm, 100 × 4.6 mm; Phenomenex, Torrance, CA) protected by a KrudKatcher Ultra HPLC in-Line Filter (Phenomenex) using an Agilent 1200 HPLC system (Agilent Technologies, Santa Clara, CA). Mobile phase A was water with 0.1% formic acid, while mobile phase B was acetonitrile with 0.1% formic acid. The gradient started with 20% B, increased to 40% B over 20 min, followed by return to the initial condition over 5 min. The solvent flow rate was 1 mL/min and the column temperature was set at 40 °C. The diode array detector was set at a wavelength of 360 nm.

LC-ESI-tandem mass spectrometry (MS^2^) analysis

A Kinetex C_18_ reverse-phase column (2.6 μm, 2.1 × 100 mm) (Phenomenex) protected by a KrudKatcher Ultra HPLC in-line filter (Phenomenex) was used for separation. Mobile phase A consisted of water with 0.1% formic acid, whereas mobile phase B consisted of acetonitrile with 0.1% formic acid. The gradient was started at 10% B and increased to 50% B over 5 min and 90% B over 10 min followed by a return to the initial condition over 5 min. The solvent flow rate was set at 0.2 mL/min and the column temperature was set at 35 °C. LTQ instrument was operated in negative ion electron spray mode at an ion spray voltage of −4.2 kV and a source temperature of 275 °C. Sheath gas flow rate was 35 arb and aux gas flow rate was 5 arb. Data acquisition was performed using Xcalibur software in selected ion monitoring scan (SIM) mode. MS conditions used to detect glucuronides are summarized in Table S2.

Figures


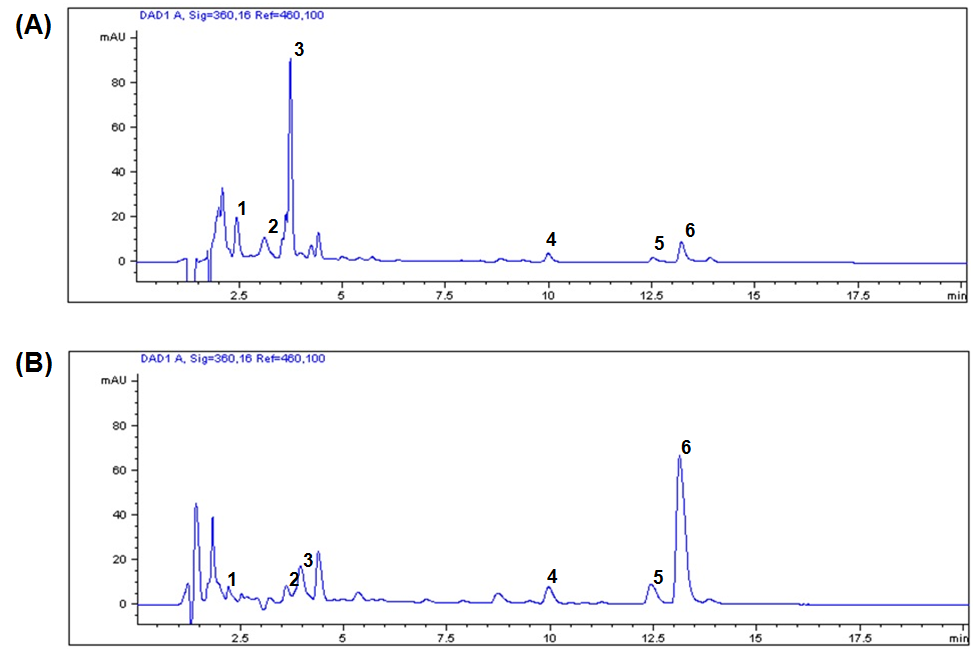


**Figure S1.** HPLC-DAD chromatogram of the *Opuntia ficus indica* fruit extract (OFIE) (**A**) and hydrolyzed OFIE (**B**) showing the major flavonoids and increase in flavonols after enzymatic hydrolysis. Rutin (1), nicotiflorin (2), narcissin (3), quercetin (4), kaempferol (5), and isorhamnetin (6). The amount of each flavonoid constituents is listed in Table S3.


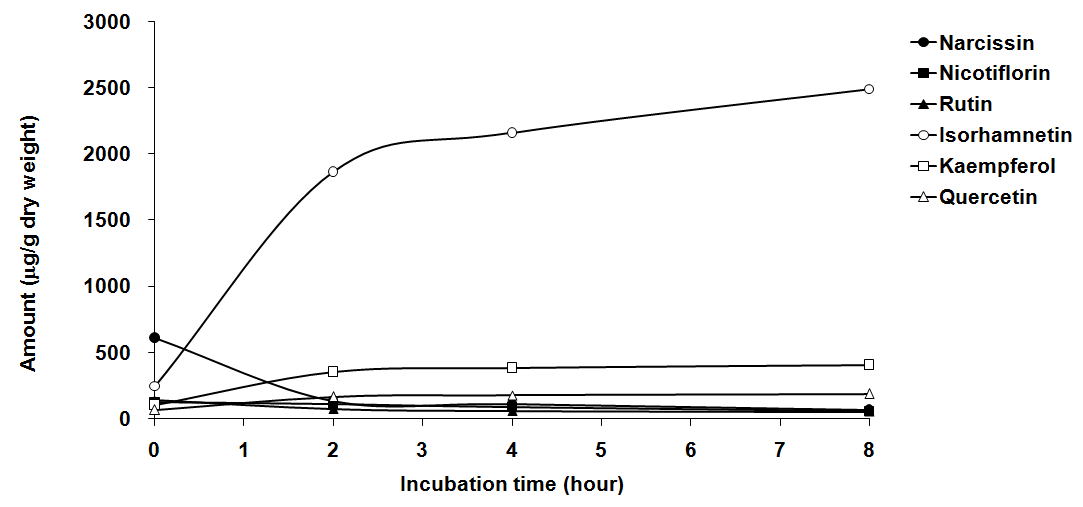


**Figure S2.** Production of flavonol aglycones by enzymatic hydrolysis of *Opuntia ficus indica* fruit extract (OFIE). OFIE was subjected to hydrolysis by incubating with cellulase and pectinase (ratio 1:4) and the hydrolyzed samples were analyzed by HPLC-DAD for flavonoid quantitation.

Tables

**Table S1.** Sequences of primers used for the detection of rat CYPs and UGTs in quantitative PCR analysis.

| **Gene name** |  | **Sequence 5′ to 3′** | **Size (bp)** |
| --- | --- | --- | --- |
| *CYP3A1* | F | TCTGTGCAGAAGCATCGAGTG | 253 |
|  | R | TGGGAGGTGCCTTATTGGG |  |
| *CYP2B1* | F | AACCCTTGATGACCGCAGTAAA | 142 |
|  | R | GTTCTTGGGAAGCAGGTACCCTC |  |
| *CYP1A2* | F | TCAACCTCGTGAAGAGCAGCA | 122 |
|  | R | CATTGAAGTTCTTAAACCTC |  |
| *CYP2C11* | F | AAAAGCACAATCCGCAGTCT | 216 |
|  | R | GCATCTGGCTCCTGTCTTTC |  |
| *CYP2D1* | F | GCTGACAAGGTCTTCCAAGG | 188 |
|  | R | ACCACCATGCGTAGGTTCTC |  |
| *UGT1A1* | F | TGGTGTGCCGGAGCTCATGTTCG | 341 |
|  | R | GTTGTATGTTTTAACCACACGCA |  |
| *UGT1A3* | F | GGCACCACTTGCTGGGCCACCTTC | 324 |
|  | R | GTAACCTGGGAATATAAGAAGAAG |  |
| *UGT1A6* | F | CCTCAGTGAACGCGGACACGAC | 335 |
|  | R | CCAGGATCACACCACAGGGCATG |  |
| *UGT1A7* | F | CAGTTGGCAGCTGGGAAAACCA | 353 |
|  | R | CTTCAAGATAGTGACAAAATATC |  |
| *UGT2B1* | F | CAGTAATTATCCCCTTCTGGAC | 346 |
|  | R | GAAAGTTAGAGCCACACAGTTC |  |
| *GAPDH* | F | GCACAGTCAAGGCTGAGAATG | 140 |
|  | R | GGTGGTGAAGACGCCAGTA |  |
| *Rplp1* | F | TAAGGCCGCGTTGAGGTG | 150 |
|  | R | GATCTTATCCTCCGTGACCGT |  |
| *Rpl13a* | F | GCAAAGATCCATTACCGGAAG | 139 |
|  | R | ACAGTCTTTATTGGGTTCACAC |  |

**Table S2.** Selected ion monitoring parameters for quantification of glucuronides by HPLC-MS^2.^

| **Parameters in MS operation** | **UGT Phenotyping reaction** | | | | |
| --- | --- | --- | --- | --- | --- |
|  | 17β-Estradiol 3-*O*-glucuronide (ESG)^a^ | Chenodeoxycholic acid 24-glucuronide (CDCAG) | 1-Naphthol-D-glucuronide (NPG) | Mycophenolic acid *O*-glucuronide (MPAG) | Zidovudine 5'-glucuronide (AZTG) |
| Capillary voltage (V) | −41 | 3–10 | −49 | −32 | −41 |
| Tube lens (V) | −122.2 | −32.5 | −12.5 | −25.5 | −122.2 |
| Analyte *m/z* transition | 447→271 | 567→391 | 319→143 | 495→391 | 442→125 |
| Retention time (min) | 9.5 | 10.9 | 9.2 | 9.8 | 6.7 |

^a^ Phenotyping reaction ESG is performed to detect the activity of UGT1A1; CDCAG, UGT1A3; NPG, UGT1A6; MPAG, UGT1A7; and AZTG, UGT2B7, respectively.

**Table S3.** Composition of *Opuntia ficus indica* (OFI) fruit extract and hydrolyzed (hdl) OFI.

| Flavonoid name | Amount in *Opuntia ficus indica* (OFIE) fruit extract (g/g dry weight) ^a^ | Amount in hydrolyzed (hdl) OFI  (g/g dry weight) |
| --- | --- | --- |
| Narcissin | 609.8 | 111.5 |
| Nicotiflorin | 123.2 | 84.6 |
| Rutin | 140.3 | 59.4 |
| Isorhamnetin | 244.7 | 2158.9 |
| Kaempferol | 103.8 | 384.1 |
| Quercetin | 66.9 | 175.0 |

^a^ The amount of individual flavonoid constituent was determined using HPLC-DAD analyses as described in Supplementary Materials Method Section.
